# Iodine level concentration, coverage of adequately iodized salt consumption and factors affecting proper iodized salt utilization among households in North Ethiopia: a community based cross sectional study

**DOI:** 10.1186/s40795-019-0291-x

**Published:** 2019-04-18

**Authors:** Abraham Aregay Desta, Usha Kulkarni, Kidan Abraha, Solomon Worku, Berhe Woldearegay Sahle

**Affiliations:** 1 Tigray Health Research Institute, Tigray, Ethiopia; 2 School of Public Health, Mekelle University, Ethiopia, Mekelle, Ethiopia; 3 Tulane University Technical Assistant Project Ethiopia, Addis Ababa, Ethiopia

**Keywords:** Iodine, Iodized salt, Proper utilization, Iodine deficiency, Ahferom District, Tigray, Ethiopia

## Abstract

**Introduction:**

Adequate iodine fortified salt is the most common and effective method of preventing iodine deficiency. Studies showed households using iodized salt (15 Parts Per Million (PPM) to 80 PPM) of iodine at household level were low in Tigray region and other regions of Ethiopia. Limited studies have conducted on utilization of iodized salt at the household level and none of them did not addressed on factors affecting to proper iodized salt utilization. The aim of this study was to determine the iodine concentration in the collected salt samples, adequately iodized salt consumption coverage and identify factors affecting to proper iodized salt utilization amongst the households of Northern Ethiopia.

**Methods:**

Community based cross-sectional designs on selected 318 household food caterers were interviewed and salt samples were accordingly collected. Data was analyzed by the SAS-9.2 statistical software package. The iodine concentrations of the salt samples were determined by using the golden standard iodometric titration technique. Logistic Generalized Estimating Equation (GEE) statistical analysis method was used to assess factors affecting proper iodized salt utilization at household level.

**Results:**

Adequately iodized salt coverage among the households was only 51 (17.5%). About 42 (14.38%) had 15 ppm (ppm) – 80 ppm, 9 (3.08%) had > 80 ppm, 188 (64.4%) had 1.1 ppm to 14.9 ppm and 53 (18.2%) had no iodine in the salt (0 ppm). Only 26 (8.9%) of the households had used iodized salt properly. Family size with Adjusted Odds Ratio (AOR) (0.82) and 95%CI [0.67, 0.92], residency of the household with AOR (2.83) and 95%CI [1.48, 5.40], the availability of iodized salt with AOR (3.90) and 95% CI [1.74, 8.7] and affordability to iodized salt with AOR (3.33) and 95% CI [1.41, 7.34] was strong predictors to proper iodized salt utilization.

**Conclusions:**

Coverage of adequately iodized salt was low. Family size, residency, availability and affordability of iodized salt were the predictors of proper iodized salt utilization. To enhance USI utilization effective inspection and regulatory measures should be taken to prevent the production and distribution of under/ over iodized salt in the market.

**Electronic supplementary material:**

The online version of this article (10.1186/s40795-019-0291-x) contains supplementary material, which is available to authorized users.

## Background

Iodine is an essential micronutrient for the regulation of physical growth and neural development. The daily requirement of iodine for adults is 150 micrograms [1]. Healthy humans require iodine as an essential component of the thyroid hormones, thyroxine and triiodothyronine. Failure to have adequate iodine leads to inadequate hormone and pituitary to produce Thyroid Stimulating Hormone (TSH) which results in goiters. Inadequate hormones affect many different parts of the body, particularly muscles, the heart, the liver, kidneys, and the developing brain in which collectively known as Iodine Deficiency Disorders (IDDs) [2].

IDDs remain as a significant public health problem in many countries [3]. About 1.88 billion people worldwide remain at risk of insufficient iodine intake [4]. Iodine deficiency is the major cause of preventable mental retardation/ brain damage in the world [1]. About 38 million newborns in developing countries every year to remain unprotected from the lifelong consequences of brain damage associated with IDDs [5]. Iodine deficiency alone can lower mean Intellectual Quotient (IQ) scores by 13.5 points [6] and a mild iodine deficiency can cause a significant loss of learning ability [7]. Ethiopia is a country with a high prevalence of IDDs. Goiter prevalence rates vary significantly from region to region in Ethiopia and in certain areas the prevalence rate may be as high as 71% [8]. Total goiter prevalence in Ethiopia was 35.8% in which 24.3 and 11.5% were palpable and visible goiter respectively. Goiter prevalence in the Tigray regional state were greater than 30% an indication of severe iodine deficiency [9].

The global prevention and control of IDDs recommends Universal Salt Iodization (USI) to a level of 30–100 PPM with any iodine compounds [10] as the most cost effective development efforts contributing to economic, social development and sustainable long term first line public health measure [11–14]. Characteristics of families not using iodized salt need to be known to expand coverage of iodized salt [15]. Households in low socioeconomic categories comprise a vulnerable subset of the population at risk of being exposed to under-iodized salt [12].

Even though the Ethiopian Ministry of Health (MOH) launched USI in Ethiopia in 1995 for long term sustainability of IDD prevention and control program [3]. Awareness of IDD problems and the benefits of iodized salt by the Ethiopian public are low [16]. To enhance iodized salt utilization level, consistent monitoring of iodine in salt at production, storage, sale and consumption level; and prevention of sale of non-iodized salt are key components of salt iodization programs [17]. Iodine is a sensitive mineral and losses might occur during improper practice [12]. Hence Quality and Standards Authority of Ethiopia, has set the iodine level to be 60–80 PPM as potassium iodate (KIO_3_) after making allowance for losses during storage and distribution. The presence of iodized salt alone is not enough, but appropriate practice at household level is mandatory towards meeting the goal of IDDs elimination [17].

Different studies showed households using adequately iodized salt at the household level were low in Tigray region and other regions of Ethiopia [18–20]. None of these studies did not addressed on factors affecting to proper iodized salt utilization at household level. Likewise, there were limited studies done on iodized salt utilization at both national and regional household levels. Therefore, the main aims of this study were to determine the iodine concentration in the collected salt samples, adequately iodized salt consumption coverage and identify factors affecting on proper iodized salt utilization among the households of Ahferom district, North Ethiopia.

## Methods

### Study design and setting

A community based quantitative cross-sectional study design was conducted from Jan 08, 2012 to Jun 16, 2012 in Ahferom district located around 200 Kilometers from Mekelle, the capital city of the Tigray regional state. At the time of the study there were about 44,194 households and 207,712 residents in the district. Tigray region is among the 9 regional states of Ethiopia.

### Eligibility criteria

Respondents who were 18 years and above and who had volunteered to interview were included in the study. Respondents who resided in the area for less than 6 months and those unable to communicate properly were excluded from the study.

### Sample size

The sample size of this study was calculated from EDHS 2005 household iodized salt coverage in Tigray region [19] with the assumption that it was close to represent households in Ahferom district. Taking 5% margin of error and 95% confidence interval of certainty (alpha =0.05), the actual sample size for the study was computed using one- sample population proportion formula as indicated below. Ten percent of the sample was added considering of the non respondents. *N* = 44,194 households.

$n=\frac{{\left({z}_{\alpha /2}\right)}^2\times pq}{d^2}$ Where, n = Sample size, z_α/2_ = Critical value 95% confidence interval = 1.96, P = proportion of being using iodized household salt = 0.25, q = proportion of not being used iodized household salt = 0.75, d (marginal error) = 0.05

$$ n=\frac{(1.96)^2\times 0.25\times 0.75}{(0.05)^2} $$

$$ n=288.12\approx 289 $$

Including 10% of non responsiveness, the final sample size was 318 households.

### Sampling technique

**Fig. 1:**
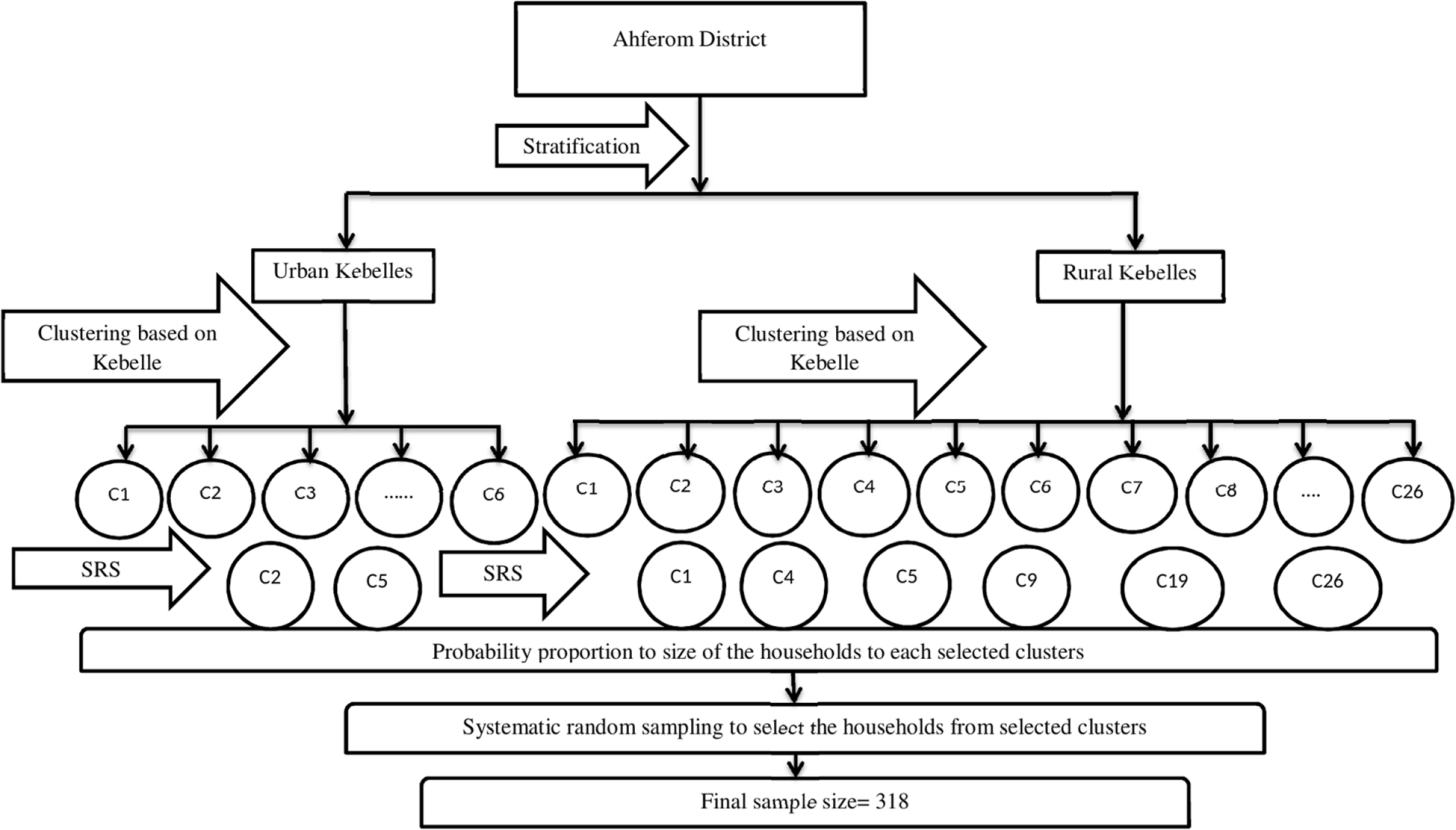
Schematic presentation of the sampling procedure

A multistage with three stage sampling process was used to ensure representative of all residents in the district. In stage 1, to confirm uniformity kebelles (smallest administrative structure) were stratified by residence type. Kebelles were considered as uniform in characteristics, hence they were considered as clusters. In stages 2, 2/6 kebelles from urban and 6/26 kebelles from rural were randomly selected by the lottery method after listing all kebelles in both urban and rural areas separately. Probability proportion to the size of the households was used to allocate number of sampled households in each of the selected Kebelles for the study. In the final stage, systematic random samplings were used to select the households from each selected Kebelles. To do that sampling interval [K] was calculated to each selected kebelles. The survey in each kebelle was started after pinning pen to indicate the direction from where to start and the first household was selected after counting households of K/2 for even interval or K/2 + 1 for the odd interval from the first household contact. And the next households were selected every K + K/2 from the first selected household to the next household until it reaches to the allocated final sample size of each kebelles (Fig. 1).

### Data collection tools and procedure

Data was collected using structured questionnaires which sought information on socio-demographic and economic variables, availability and accessibility of iodized salt, practice of salt utilization, concentration of iodized salt, Knowledge and attitude regarding to iodized salt and IDDs. The questionnaires were adapted from different studies taking into account the local situation of the study area [10, 21] (Additional file 1). The collected salt samples were tested by using an iodometric titration technique to measure the iodine concentration. This process was done in Tigray Health Research Laboratory.

The salt samples were collected from the top, middle, and bottom of the pack (bag) using a moisture free, clean plastic container with cover. The samples were labeled with the following information during collection: Date of sampling, name of the Kebelle, and house number. During the visits, we first explained the aims of the study to household food caterers. After obtaining informed consent, each participant was interviewed by trained data collectors (diploma nurses). In addition, three public health officers were recruited as supervisors. After completion of the interview to each household food caterers, they were requested to provide three teaspoons (15 g) of consumption salt for iodine concentration test.

### Operational definitions

Adequately iodized Salt: It is a salt that is fortified with the iodine which is > = 15PPM.

Improper utilization: practicing at least one practice that reduces the iodine content or < 15 PPM Iodine concentration in the salt.

Food caterer: Household member responsible for cooking in most of the time.

Self-report of “Yes, I used iodized salt”: a food caterer who knows and used iodized salt within 24 h.

### Data quality assurance

To ensure data quality and consistency of the measurement tool, the questionnaire that was in Tigrigna was translated back to English. About 5% of the total participants of the study were pre-tested by similar households to check any discrepancy. Data was collected under close supervision and data was checked for completeness daily by the principal investigators. The quality of the test of the iodometric titration technique was checked to positive and negative controls.

### Data management and analysis

Data was entered to SPSS Version 16.0 and exported to SAS version 9.2 for analysis. Knowledge was assessed by asking a range of questions about IDD, iodized salt and marking the correct answers of subjects out of a hundred. Average knowledge scores 50% or less was labeled as “poor knowledge” [10]. Using a Likert scale attitudes was assessed with five possible responses. The responses was labeled “favorable” or “unfavorable” as follows; For positive statements, responses including strongly agree and agree were labeled as “favorable” and disagree, strongly disagree and uncertain were labeled as “unfavorable”. For negative statements, those who responded “strongly agree”, “Agree” and uncertain was labeled as “unfavorable” and strongly disagree, disagree was labeled as “favorable” response. If the average attitude scores were greater than 50% it was considered as a favorable attitude [11].

Descriptive statistics were done to determine the proportion of households using adequately iodized salt, socio demographics and concentration of iodine in the salt. An inter observer variation of the iodized salt between self report of the respondents and iodometric titration was measured by using kappa statistics. The Kappa agreement was interpreted according to the scale [22]. Specificity, sensitivity, positive predictivity, negative predictivity and predictive validity of self-report on the use of iodized salt were calculated to check its validity with iodometric titration.

The outcome variable was a dichotomous outcome (1 = proper iodized salt utilization and 0 = improper iodized salt utilization). The analytic approach to modeling this type of data was the logistic generalized estimating equation (GEE), which takes into account the correlated nature of the responses. The order of responses within a cluster was arbitrary; therefore it was considered exchangeable and independent correlation structures [23]. The specified probability distribution was binomial with logit link function and the working correlation matrix structure was exchangeable (with the small Quasi likelihood under Independent Criterion (QIC)). The covariance matrix was robust estimator, and the scale parameter was Person chi-square (χ2). The main effect was the term used to build the reported model, and Kernel was specified for the log quasi-likelihood function. Bivariate logistic regression was used to see the strength of associations and factors that was found significant at *p*-value<=0.05 at bivariate analysis was entered to multivariate logistic regression to specify the independent predictors. Odds ratios (OR) were calculated to determine the strength of associations of the independent variables with the outcome variable at 95% Confidence Interval (CI).

Interactions of variables were assessed at p-value <= 0.05 and confounding of variables were assessed by backward and forward elimination and any variable which had > 20% change of coefficient of the parameters between the reduced and full model was considered as confounder [23]. Similarly Collinearity was checked by Variance Inflation Factor (VIF) and If VIF was greater than 10 it was considered as collinear and removed from the model.

## Results

### Socio demographics of the household respondents

Three hundred eighteen (318) food caterers were interviewed in the selected households in Ahferom district. The response rate was 100%. Out of the selected households 230 (72.3%) were from rural areas and around 220 (69.2%) of the households were headed by males. Two hundred forty seven (77.7%) of the households have children less than 12 years of age. Around 262 (82.4%) of the occupation of the food caterers were farmers followed by Merchants 25 (7.9%). The source of income for the majority of households was farming 252 (79.2%) followed by trading 28 (8.8%). Most of the households had a family size from 1 to 4 and the average family size of the households was around 5.44 with a standard deviation of 2.2 and a range of 1–10.

Regarding the education level of the head of the households 134 (42.4%) was illiterate and most of the food caterers were illiterate 188 (59.1%). Similarly, most of the households 224 (75.42%) had a monthly income level below 500 Birr, 49 (16.5%) had a monthly income level between 501 to 1000 Birr (Table 1).

**Table 1:** Demographic Characteristics of the respondents of the households interviewed for proper iodized salt utilization in Ahferom District, North Ethiopia, 2012 (*n* = 318)

variables	Frequency	Percentage (%)
Household esidence		
Urban	88	27.7
Rural	230	72.3
Sex of the head of the household		
Female	98	30.8
Male	220	69.2
Children below 12 years		
No	71	22.3
Yes	247	77.7
Occupation of food caterers		
Farmer	262	82.4
Merchant	25	7.9
Spouse	10	3.1
Governmental employee	7	2.2
Day laborer	7	2.2
Others	7	2.2
Source of household income		
Farming	252	79.2
Trading	28	8.8
Trading and Farming	10	3.1
Daily employee	7	2.2
Governmental	6	1.9
Others	15	4.7
Household monthly income level in Birr		
< 500	224	75.4
501–1000	49	16.5
1001–1500	13	4.4
1501–2000	8	2.7
Above 2000	3	1.0
Educational level of head of HH		
Illiterate	134	42.4
Read and write	46	14.6
Elementary School	116	36.7
Secondary school	13	4.1
College/ University	7	2.2
Educational level of food caterers		
Illiterate	188	59.1
Read and write	22	6.9
Elementary School	92	28.9
Secondary school	11	3.5
College/ University	5	1.6
Age of food caterers		
Independent age group	299	94.3
Dependent age group	18	5.678
Family size		
1–4	124	39.2
5–6	79	25.0
7–10	113	35.8

### Adequately iodized salt coverage among the households

Out of the 318 respondents, about 168 (52.83%) reported that as they have used iodized salt while the rest reported that as they did not use iodized salt. There was a larger discrepancy when the iodine content was tested by iodometric titration and self-reported use of iodized salt among 292 households. As per the self reported responses who said “yes, I use iodized salt”; 112 (72.26%) had not used iodized salt and 43 (27.74%) had used iodized salt according to the iodine test. In contrast to the self reported responses: who said “I don’t use iodized salt”, about 129 (94.16%) had not used iodized salt and only 8 had used iodized salt according to the iodine test. The Pearson chi square is significant 2-sided at *p*-value< 0.01 with alpha level = 0.05. Overall, adequately iodized salt coverage among the households was 51 (17.5%) as shown in (Table 2).

**Table 2:** Self-reported use of iodized Salt cross tabulated with iodometric test for proper iodized salt utilization in Ahferom District, North Ethiopia, 2012 (*n* = 292)

Self report use of Iodized salt	Iodomethric test	Total		
	Iodized salt	Not iodized		χ2 significance level
Yes	43	112	155	0.001
No	8	129	137	
Total	51	241	292	

Kappa was done to estimate, measure of agreement between self report on the use of iodized salt and iodometric test. The Observed and expected agreement were 0.59 and 0.48 respectively. Whereas Kappa (K) was 0.21 95% [0.13–0.29]. In health research if Kappa (K) is 0 < K < 0.40 it is marginal/poor. Therefore, this shows that prediction of perceived self report on the use of iodized salt was poor. Sensitivity and specificity of the perceived self report on the use of iodized salt were 0.84 and 0.535 respectively. Whereas positive and negative predictivity of the perceived self report on the use of iodized salt was 0.277 and 0.942 respectively. About 27.7 and 94.2% of the perceived self reports had predicted correctly the use of iodized salt and non iodized salt respectively. Similarly about 58.9% of the self reports had combined ability to correctly predict the use of iodized and non iodized salt.

### Concentration of iodine in the salt collected from the households

**Fig. 2:**
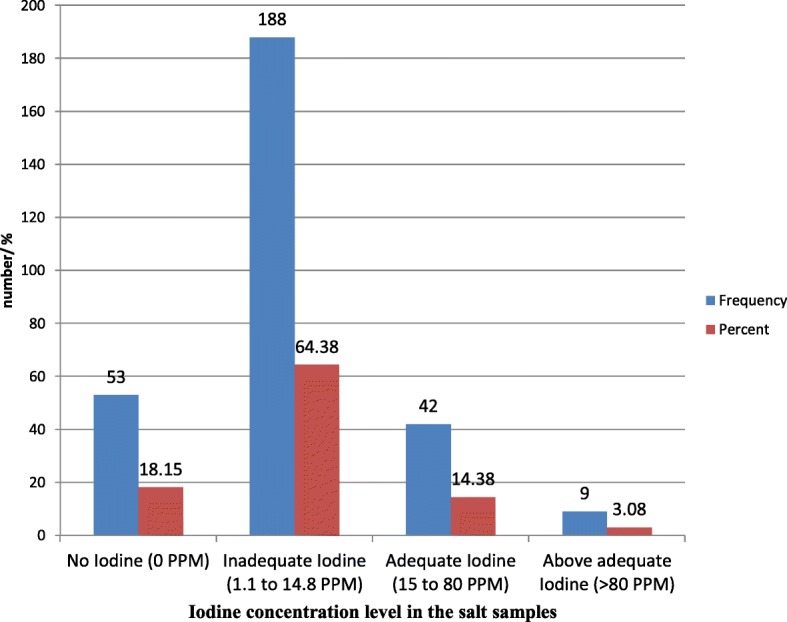
Iodine concentration level in the salt samples collected from the households of Ahferom District, North Ethiopia, 2013 (*n* = 292)

Two hundred ninety two (92%) of the household salt samples were tested for the presence of iodine using iodometric titration technique. There were 26 household salt samples not tested for iodine concentration because of inadequate salt sample (less than 10 g). The iodometric titration technique showed that 53 (18.15%) household salt samples had no iodine in the salt (0 PPM), 188 (64.38%) had a low concentration ranging from (1.1 PPM to 14.8 PPM), 42 (14.38%) had adequate iodine concentration (15PPM- 80PPM) and only 9 samples had iodine concentration above adequate (>80PPM) (Fig. 2).

### Factors affecting proper iodized salt utilization among the households

#### Bivariate analysis

Out of the 318 households only 89 (27.99%) had proper practice to iodized salt utilization based on their response. However, households using proper iodized salt utilization (in combination of concentration and practice) were only 26 (8.9%). Urban residence Crude Odds Ratio (COR) = 6.22; 95% CI (3.05, 12.66), Family size COR = 0.70; 95% CI (0.62, 0.80), Education level of Food caterers with at least read and write COR = 2.09; 95% CI (1.23, 3.54], Amount salt in kg buy at a time COR = 0.64; 95% CI (0.45, 0.89), Availability of iodized salt COR = 14.47; 95% CI (4.95, 42.27) and Affordability of iodized salt COR = 8.33; 95% CI (2.99, 23.21) were significant variables in bivariate analysis (Table 3). There was no significant interaction among the variables in the bivariate analysis.

**Table 3:** Bivariate and Multivariate analysis of proper iodized salt utilization among the households in Ahferom District, north Ethiopia, 2012 (n = 292)

Variables	Iodized salt utilization			COR [95% C.I]	AOR[95%CI]
	Improper n[%]	Proper n[%]	Total[n]		
Sex head of household					
Female	77 (89.5]	9 (10.5)	86	1[ref]	
Male	189 (91.7)	17 (8.3)	206	0.77[.26, 2.29]	
Residency					
Rural	204 (97.1)	6 (2.9)	210	1[ref.]	
Urban	62 (75.6)	20 (24.4)	82	6.22 [3.05, 12.66]**	2.83 [1.48, 5.4]*
Age of food caterers				.95[.89, 1.02]	
Family size				.70[.62, .80]**	0.82 [0.67, 0.92]*
Children< 12 years				.74[.52, 1.06]	
Occupation of food caterers					
Farmer	222 (91.7)	20 (8.3)	242	1[ref.]	
Others	44 (88.0)	6 (12.0)	50	1.51[.81, 2.84]	
Income source					
Farming	215 (91.9)	19 (8.1)	234	1[ref.]	
Others	51 (87.9)	7 (12.1)	58	1.55[.98, 2.45]	
Household monthly income					
< =500 birr	185 (91.1)	18 (8.9)	203	1[ref.]	
> =500 birr	81 (91.0)	8 (9.0)	89	1.39[.63, 3.09]	
Education head of household					
Illiterate	111 (94.1)	7 (5.9)	118	1[ref.]	
At least read & write	155 (89.1)	19 (10.9)	174	1.94[.78, 4.86]	
Education of food caterers					
Illiterate	161 (93.6)	11 (6.4)	172	1[ref.]	
At least read & write	105 (87.5)	15 (12.5)	120	2.09 [1.23, 3.54]*	
Knowledge cat					
Poor	139 (95.2)	7 (4.8)		1[ref.]	
Good	127 (87.0)	19 (13.0)		2.97[.83, 10.69]	
Attitude					
Unfavorable	110 (91.7)	10 (8.3)	120	1[ref.]	
Favorable	156 (90.7)	16 (9.3)	172	1.13[.29, 4.32]	
Source of buying					
Out of shop	165 (94.8)	9 (5.2)	174	1[ref.]	
Shop	101 (85.6)	17 (14.4)	118	3.09[.70, 13.69]	
Duration buying salt					
Above 1 month	47 (94.0)	3 (6.0)	50	1[ref.]	
< =1 month	219 (90.5)	23 (9.5)	242	2.22[.45, 10.99]	
Amount salt in kg buy at a time				.64[.45, .89]*	
Availability of iodized salt					
No	192 (98.0)	4 (2.0)	196	1[ref.]	
Yes	73 (76.8)	22 (23.2)	95	14.47 [4.95, 42.27]**	3.90 [1.74, 8.70]**
Affordability of iodized salt					
No	135 (97.8)	3 (2.2)	138	1[ref.]	
Yes	131 (85.1)	23 (14.9)	154	8.33 [2.99, 23.21]**	3.22 [1.41, 7.34]*

*COR* crude odds ratio

*AOR* adjusted odds ratio

*(*p*-value < 0.05)

**(*p*-value <= 0.001

#### Multivariate analysis

The multivariate analysis showed Urban residence Adjusted Odds Ratio (AOR) = 2.83; 95% CI (1.48, 5.4), Family size AOR = 0.82; 95% CI (0.67, 0.92), Availability of iodized salt AOR = 3.90; 95%CI (1.74, 8.70) and Affordability of iodized salt AOR = 3.22; 95%CI (1.41, 7.34) were significant variables in multivariate analysis (Table 3).

## Discussion

The main aim of this study was to assess adequately iodized salt coverage, iodine concentration in salt and factors affecting proper iodized salt utilization among the households of Ahferom District, North Ethiopia. This in-depth community-based study revealed that 52.83% of the respondents have self-reported as they use adequately iodized salt, however, only 17.5% of the households were using adequately iodized salt when tested using an iodometric titration technique. The kappa agreement of iodometric titration test and validity of predicting adequately iodized salt by using perceived self report was poor. This indicates either there was a problem in identification of iodized salt during the purchasing or there might be a loss of iodine more than expected during storage and/ or transportation of the iodized salt in the whole sellers, shops and others where salt was sold.

The coverage of adequately iodized salt among the households based on the test was 17.5%. This was much farther to the WHO target of 90% coverage [12]. This coverage was also very low when compared to the studies conducted in India in 2005 and to the study conducted in South Africa in 2001 which had iodized salt utilization 65 and 63% respectively [6, 12]. It was four folds lower if it was compared with the study conducted in Ghana in Bia district in 2011 [24]. It is also slightly lower as compared to the National studies conducted in 2005 (20% coverage) in Ethiopia [25]. And it was better compared to the EDHS 2000 (10% coverage) report with children under 5 years’ households of Tigray region [18]. However, it was lower coverage compared as per EDHS 2005 (25%) and 2011 (22.3%) report in Tigray but similar to Ethiopia as per EDHS 2011 (15.4%) [19, 20].

Similarly, this coverage was very low (greater than fivefold lower) as compared to the study conducted in 2002 in Shebe town, southwestern Ethiopia, which showed 92.7% of the household had used iodized salt [10]. The finding of this study was also lower than the study done in Gondar North west Ethiopia in 2012, which was 28.9% coverage [26]. Another study conducted in Bensa district Sidama, Ethiopia indicated adequately iodized salt (≥15 ppm) was found in 45.2% rural and 65.0% urban households [27].

The reason why the coverage of households using iodized salt in this current study was lower than the global and regional levels might be due to the influence of low national coverage or distributional barriers to different parts of the country as well as poor community awareness on prevention of IDDs and the benefits of iodized salt [16]. Reasons have been given for the failure of most developing countries to achieve 90% iodized salt utilization among the households. These were political factors and logistical problems in the production and distribution of iodized salt [25]. The other reason might be poor enforcement practices by regulatory agencies, infrequent to no monitoring and evaluation. Just because a certain target is achieved in one evaluation by no means assures that future results will be consistent. Hence continual program monitoring and cooperation of regulatory enforcement agencies are essential to ensuring the adequacy of iodine fortification levels. On the other hand, Ethiopia is not major salt producing country [28] and even there might be inadequacies in regulation of the fortified salt produced within the country. This could have an influence to decrease coverage of adequately iodized dietary salt at household level.

The most effective way to control, IDD is through salt iodization, WHO, UNICEF, and ICCIDD recommended that iodine should be added to salt a concentration of 20–40 PPM depending on salt intake [29]. About 17.5% of the salt sample taken from the households in this study had iodine concentration above 15 PPM. This was fourfold lower even if it was compared to to the iodine concentration above 25 PPM, to the study that was conducted in Bia district, Ghana in 2011 [24]. When this study was also compared to the study conducted in Shebe town, Western Ethiopia [10]; the concentration of iodine >15PPM were even lower than the concentrations between 60 PPM- 80PPM. This shows that there was a huge gap of iodized salt utilization in this study area. These differences might be due to poor regulation activities against non iodized salt in the market, where salt was sold.

According to the Quality and Standards Authority of Ethiopia, has set the iodine level shouldn’t exceed above 80 PPM as potassium iodate (KIO_3_) after making allowance for losses of iodine during storage and distribution [3]. However, there were salt samples collected from 9 households that had an iodine concentration above 80 ppm in this study. This shows that there might be iodized salt producers not having any inspection procedures by the Authorities.

Proper utilization of iodized salt among the households was 18% less likely lower as family size increase by one other factors held constant. This OR could be as low as 0.67 and as high as 0.92 at 95% CI. Even though there were no other studies that support for this finding, as the family size increase economic constrain might increase, which leads to reduce in the purchasing of iodized salt to the household members since it is slightly higher in its cost compared to the non iodized salt in the market.

The odds of proper iodized salt utilization among households resided in urban was 2.83 times higher than households that had resided in rural other factors held constant. The OR ranges from 1.48 to 5.40 at 95% CI. There were no previous studies that show the strength of association with regard household residency. However EDHS 2011 described that urban household is more likely to use iodized salt than rural households [20]. This difference might be urban residents might have better access to mass media that motivates them to utilize iodized salt properly. In addition to that there were higher in education status in urban compared to the rural food caterers which can have better knowledge and practice to iodized salt utilization.

Households that have availability of iodized salt have utilized iodized salt properly 3.90 times the odds of those households which didn’t have availability to iodize salt other factors held constant. The 95% CI was from 1.74 to 8.70. This finding in line with most salt produced in Ethiopia was non iodized, iodized salt was estimated to be less than 5% in 2009 [28]. The main reasons for such insufficient accessibility might be many and various, both at the level of sellers and consumers. Various demographic, societal and economic factors may have influences on iodized salt utilization practice [30]. In many cases it has been observed that awareness of the use of adequately iodized salt as a preventive measure against IDD exists but the access to iodized salt is limited. In most cases the restriction to iodized salt utilization was because of iodized salt was not available [25].

The odds of proper iodized salt utilization among households that can afford the cost of iodized salt was 3.22 times higher than households that couldn’t afford the cost of iodized salt all other things being equal. The estimated 95% CI was from 1.41 to 7.34. There are descriptives that have aligned with this study. Socio economics factors are the most contributing factor to IDD because least developed countries are more affected [31]. Study in Ethiopian households revealed those highest wealth Quintiles were twice as likely to use iodized salt as households in the lowest two wealth Quintiles. Those with highest wealth quintile might have a capacity to afford iodized salt as compared to that lowest quintile. This restriction of iodized salt was sometimes due to affordability [25]. There were evidences supported for this finding by researches conducted in low and middle income countries [32]. It have Confirmed better practices of iodized salt has a relationship with the price of the salt [33]. A similar evidences have reported that excess cost of iodized salt might be a barrier in preventing IDDs. This forces people not to purchase and use the iodized salt [34].

### Strengths and limitations of this study

The strengths of this study were: Iodine concentration in salt was determined by quantitative golden standard iodometric titration technique, statistical analysis and modeling was done by logistic GEE, which can handle intra class correlation within the clustered data and this study have used to combine both household practice and quantitative concentration of iodine in salt as the outcome variable. However, this study was not without limitation; the study was done in one region and the findings may not generalize to national levels. In addition to that design effect was not considered during sample size determination and the cross-sectional study design limits the factors to establish temporal relationship; hence inference of causation is not possible.

## Conclusions

This study concluded that the coverage of adequately iodized salt among the households was low. Most of the households were using below adequate level of iodine concentration in the salt. There were households salt samples iodized above the national standard. Family size, affordability of iodized salt, availability of iodized salt in the market and household residency were the main predictors to proper iodized salt utilization. This study points importance of routine monitoring and evaluation of USI. To prevent IDDs and enhance USI utilization effective inspection and regulatory measures should be taken at the manufacturers, whole sellers and retailers to prevent the production and distribution of under/ over iodized salt in the market.

## Additional file

Additional file 1: Questionnaire English version. This file shows an excerpt of the questionnaire for the iodine concentration, adequately iodized salt coverage and factors affecting proper iodized salt utilization amongst households in Northern Ethiopia. All questions and data codes are given, together with the data types. (PDF 26 kb)

## Abbreviations

CNI: Condition Index
CSA: Central Statistical Agency
EDHS: Ethiopian Demographics & Health Survey
GEE: Generalized Estimating Eq.
HH: Head of Household
ICC: Intra Cluster Correlation
ICCIDD: International Council for the Control of IDD
IDD: Iodine Deficiency Disorder
IQ: Intellectual Quotient
K: Kappa
KAP: Knowledge Attitude and Practice
KG: Kilo Gram
KM: Kilo Meter
MOH: Ministry Of Health
MU: Mekelle University
PPM: Parts Per Million
QIC: Quasi likelihood under Independent Criterion
SNNPE: Southern Nation Nationalities and Peoples of Ethiopia
SPSS: Statistical Package for Social Sciences
TSH: Thyroid Stimulating Hormone
TUTAPE: Tulane University Technical Assistant Project Ethiopia
UNICEF: United Nations Children’s Fund; USI: Universal Salt Iodization
WHO: World Health Organization

## Authors’ information

AD, MSc in Biostatistics and health informatics, public health researcher in Tigray Health Research Institute; UK, PhD in Nutrition, instructor in Mekelle University; BS, MPH and PhD, instructor in Mekelle University; KA, MPH, instructor in Mekelle University; SW, MD, MPH, Worked in TUTAPE
