# Supplementary material for: Iodine level concentration, coverage of adequately iodized salt consumption and factors affecting proper iodized salt utilization among households in North Ethiopia: a community based cross sectional study
Source: BMC Nutr. 2019 Apr 18;5:28. doi: 10.1186/s40795-019-0291-x (PMC7050811; doi:10.1186/s40795-019-0291-x)
Supplement: Supplementary file 1 — Questionnaire English version. This file shows an excerpt of the questionnaire for the iodine concentration, adequately iodized salt coverage and factors affecting proper iodized salt utilization amongst households in Northern Ethiopia. All questions and data codes are given, together with the data types. (PDF 26 kb) [file 40795_2019_291_MOESM1_ESM.pdf]

S1: Questionnaire English version

Questionnaires and checklists on Iodine concentration, coverage of adequately iodized salt and factor affection on proper iodized salt utilization

Part I. Socio demographic characteristics of the household

**No**	**Questions and filters**	**Response coding Categorization**	**Skip**
101	Area Identification	1. Kebele __________ Kebele Code_____<br>2.Gote (Cluster) Name _______<br>3.Cluster #_________<br>4. code household_______if any	
	Residence of the household	1. Rural<br>2. Urban	
102	Sex of the head of the household	1. Male_____<br>2. Female____	
103	Age of the head of the household in years	[--------]	
104	Age of the housewife	[……..]	
105	What is your family size within the household?	|___|	
106	How many children under 12 in the household	[……….]	
107	Occupation of the house wife (*multiple answers allowed)*	1.Farmer____<br>2. Merchant___<br>3. Governmental_____<br>4.NGO______<br>4. Daily worker_____<br>5. others_______ specify_________	
108	What is the source of income for the household? (*multiple answers allowed)*	1.Farmer____<br>2. Merchant___<br>3. Governmental_____<br>4.NGO______<br>4. Daily worker_____<br>5. Others_______ specify_________	
109	Income level per month in Eth birr	[-----]	
110	What is the educational Status of the head of the households?	1. Illiterate----------<br>2. Read and write -----<br>3. Elementary school----<br>4. High school----<br>5.Higher institution------<br>6. Others------- specify----------------	
111	What is the educational Status of the housewife?	1. Illiterate<br>2. Read and write	

		3. Elementary school<br>4. High school<br>5.Higher institution<br>6. Hthers------- specify----------------	

Part ii. Knowledge about Iodine and Iodized salt

No	Questions and filters	Response coding Categorization	Skip
201	Do you know what iodine is?	1.Yes----- go to ques # 202<br>2.No------ go to ques # 211<br>3.I don't know------ go to ques #211	
202	If yes what is iodine?	1.Mineral-------<br>2.Vitamin------<br>3.Protein------<br>4.Carbohydriate-----<br>5.Others-------<br>Specify---------	
203	Do you know iodine rich foods?	1.Yes-----<br>2.No------<br>3.I don't know------	
204	If yes tell me iodine rich foods.	[……………………………….]	
205	Do you know iodine deficiency disorders?	1.Yes-----<br>2.No------<br>3.I don't know------	
206	If yes, would you tell me one iodine deficiency disorders?	[…………………………………….]	
207	Who are at risk for iodine deficiency? (*multiple answers allowed)*	1.Children------<br>2.Elder----------<br>3.Pregnant Women-----<br>4.Adolescent------------<br>5.Men-------------<br>6.Women--------<br>7.Others -----------<br>Specify…………	
208	Do you know how to prevent iodine deficiency disorders?	1.Yes-----<br>2.No------<br>3.I don't know------	
209	If yes what are the prevention mechanisms you know?	1.Iodized oil---------<br>2.Iodized salt--------<br>3.Sea foods-------<br>4.Iodized water-----<br>5.Others-----------specify---------	
210	From whom did you get the information of	(Y = yes, N = no) (Y N)	

	prevention and control of IDDs?(*multiple answers allowed)*	1.Radio……………………1 2<br>2. Television.……………...1 2<br>3.Newspaper/magazine……1 2<br>4.Pamphlet/poster…....……1 2<br>5.Health Worker….……….1 2<br>6.Community events………1 2<br>7.Family/friends……………1 2<br>9.Health extension worker...1 2<br>10.Others…..………………1 2<br><br>(Specify)-----------------------	
211	What kind of salt do you use?	1. Refined…………..<br>2. Coarse -------------<br>4. Others ... ………<br>(please specify)-------------	
212	Do you use iodized salt?	1.Yes-----(go to quest no 213)<br>2.No-------(go to quest no 214)<br>3.Don't know---(go to quest no 214)	
213	If yes why do you choose iodized salt?	1.For health ---------<br>2.I don't know------<br>3. Testy ……..<br>3.Others -------------<br>Specify-------------	
214	If no why don't you choose iodized salt	1.it looses salty taste---------<br>2. other-------------<br>Specify-------------	
215	If you are using iodized salt how you did hear about iodized salt and its importance? (*multiple answers allowed)*	(Y = yes, N = no) Y N)<br>1.Radio……………………1 2<br>2. Television.……………...1 2<br>3.Newspaper/magazine……1 2<br>4.Pamphlet/poster…....……1 2<br>5.Health Worker….……….1 2<br>6.Community events………1 2<br>7.Family/friends……………1 2<br>9.Health extension worker...1 2<br>10. Others…….……………1 2<br>(Specify)……………………	
217	Do you know iodized salt is sensitive to temperature, sun light and humidity.	1.Yes-----<br>2.No------<br>3.I don't know------	
Part iii. Attitude about iodized salt			

301	In adequate salt intake is good for your health.	1. Strongly agree 2. Agree<br>3.Uncertain 4.disagree 5. Strongly disagree	
302	Resistance from you and member of your community against purchasing iodized salt may affect the health of you, your family and your community.	1. Strongly agree 2. Agree<br>3.Uncertain 4.disagree 5. Strongly disagree	
303	Adding salt to the food of infant/children is good.	1. Strongly agree 2. Agree<br>3.Uncertain 4.disagree 5. Strongly disagree	
304	Adding iodized salt to the food of pregnant mothers and lactating mothers is not good.	1. Strongly agree 2. Agree<br>3.Uncertain 4.disagree 5. Strongly disagree	
305	There is no harmful effect in children, lactating and pregnant mothers if they take insufficient iodine.	1. Strongly agree 2. Agree<br>3.Uncertain 4.disagree 5. Strongly disagree	
306	Influence on the decision to use iodized salt by friends and health workers are useful for you and your family.	1. Strongly agree 2. Agree<br>3.Uncertain 4.disagree 5. Strongly disagree	
**Part iv. Practice of iodized salt**			
401	Do you use iodized salt?	1.Yes-----<br>2.No-------<br>3.Don't know---------	
402	Do you buy packed salt?	Yes-------------(go to questi no 403)<br>No-------------(go to quest no 404)<br>I don’t know…(go to quest no 404)	
403	If yes how is the salt packed?	1.plastic------<br>2. thick plastic bag------<br>3. glass/ bottle------<br>4.Other------specify	
404	Is your storage of salt free from sun light, moisture and high temperature?	1.yes<br>2. no<br>3. I don’t know	
405	Do you add table salt?	1. Yes ……<br>2. No …….<br>3. I don’t know	
409	When do you usually add salt to your	1. Before cooking……….	

	food?	2. During cooking……….<br>3. After cooking…………<br>4. Other………specify …………..<br>5. don't know[------------------------]	
v. Accessibility and availability of iodized salt			
501	Where is your source of buying salt?	1.Market-----------<br>2. shop-------------<br>3. Super market-----<br>3.Others-----------<br>Specify-------------	
502	How often do you usually buy salt?	1.Weekly------<br>2. Monthly ----<br>3. Yearly--------<br>4. Others-------<br>5. Specify------	
503	How much do you usually buy at a given time? (in kgs)	[-------------]	
504	How much salt does your family consume per month in KG?	[----------------]	
505	Do you get as you want iodized salt in the in the place where it sold?	1.Yes<br>2.No<br>3. I don't know……	
506	Is there both iodized and non iodized salt in the market?	1.Yes------------(go to quest no 507)<br>2.No-------------(go to quest no 601)<br>3. I don't know--(go to quest no 601)	
507	If yes is there a difference in cost?	1.Yes----------------<br>2.No-----------------<br>3. I don't know-------	
508	If the price of iodized salt is a little bit more expensive than regular salt can you afford to buy the iodized salt?	1.Yes----------------<br>2.No-----------------<br>3. I don't know-------	
**Vi. Check list for iodine concentration in the salt.**			
601	Ask respondent three teaspoonful of cooking salt:<br>Test salt for iodine<br>Record parts per million (ppm)	1.0 ppm (no iodine)……………….1<br>2.Less than 15 ppm………………..2<br>3.More than 15 ppm………..…….3<br>4.No salt in the house……………..4<br>5. Salt not tested, specify reason……	

This is all my questions and Thank you for your response.
